# Cost-utility of behavioural activation for mitigating psychological impacts of COVID-19 on socially isolated older adults with depression and multiple long-term conditions compared with usual care: results from a pragmatic randomised controlled trial

**DOI:** 10.1136/bmjment-2024-301270

**Published:** 2025-01-19

**Authors:** Han-I Wang, Simon Gilbody, Elizabeth Littlewood, Kalpita Baird, David Ekers, Dean McMillan, Della Bailey, Carolyn Chew-Graham, Peter Coventry, Caroline Fairhurst, Catherine Hewitt, Steve Parrott

**Affiliations:** 1 Health Sciences, University of York, York, UK; 2 Hull York Medical School, Hull, UK; 3 Tees Esk and Wear Valleys NHS Foundation Trust, Darlington, UK; 4 School of Medicine, Keele University, Keele, UK

**Keywords:** COVID-19, Depression, Adult psychiatry

## Abstract

**ABSTRACT:**

**Background:**

Depression alongside multiple long-term conditions (MLTCs) in older adults poses a critical public health challenge, worsening physical and mental health and increasing healthcare costs. COVID-19 restrictions further exacerbated these impacts. Behavioural activation (BA) shows promise as a remote intervention for depression during isolation, but its cost-effectiveness for depressed, socially isolated older adults remains uncertain.

**Objective:**

This study aimed to assess the cost-utility of BA versus usual care for older adults with depression and MLTCs during COVID-19 restrictions.

**Methods:**

A randomised controlled trial recruited and randomised individuals aged 65 and over with depressive symptoms and MLTC (n=435) to either the BA intervention or usual care. Costs were measured from the perspective of the National Health Service and personal social services. Quality-adjusted life years (QALYs) were measured using the EQ-5D-3L at baseline, and 1, 3 and 12 months postrandomisation. Incremental cost-effectiveness ratios were calculated, with uncertainty addressed through non-parametric bootstrapping. Sensitivity analyses were conducted to assess the robustness of the primary analysis.

**Findings:**

Primary analysis indicated that BA generated a small cost-saving (£62.34 per older adult; 95% CI: −£120.44 to £239.70) while QALY improvements remained unchanged (0.007; 95% CI −0.036 to 0.022) compared with usual care. The probability of BA being the preferred option is 0.71. Sensitivity analyses supported the primary analysis findings, confirming their robustness.

**Conclusions and clinical implications:**

Compared with usual care, BA demonstrated a slight cost reduction while maintaining QALY improvement. The findings provide promise for BA interventions for older people with depression and MLTCs facing isolation.

**WHAT IS ALREADY KNOWN ON THIS TOPIC**

- Depression comorbid with multiple long-term conditions (MLTCs) is a significant public health issue, particularly among the ageing population. This combination worsens health, reduces quality of life, increases mortality and raises healthcare costs. Restrictions including shielding and social distancing during the COVID-19 pandemic exacerbated these challenges by increasing loneliness and social isolation, further impacting both mental and physical health. Behavioural activation (BA), an evidence-based treatment that increases engagement in positive activities, has proven effective in treating depression and symptoms associated with social isolation in older adults, making it particularly suitable for situations such as COVID-19 restrictions. However, the cost-effectiveness of BA for older adults with depression comorbid with MLTCs or those living in isolation has not been explored.

**WHAT THIS STUDY ADDS**

- This study assesses the cost-utility of remotely delivered BA compared with usual care for socially isolated older adults with depression and MLTCs during COVID-19 restrictions, filling a gap in the existing literature. The economic evaluation results indicate that BA slightly reduced costs while maintaining quality-adjusted life year improvements, with a 0.71 probability of being cost-effective. These findings are consistent with sensitivity analyses, affirming BA as a preferred option.

**HOW THIS STUDY MIGHT AFFECT RESEARCH, PRACTICE OR POLICY**

- The findings support the promise of remotely delivered BA interventions for managing socially isolated older adults with depression and MLTCs. Although the cost reduction is small, the potential savings could be significant (exceeding £75 million) when scaled nationally, especially during public health crises like COVID-19. These results are useful for future pandemics and policy decisions about managing depression and MLTCs in socially isolated older adults.

## Background

 Depression comorbid with multiple (two or more) long-term conditions (MLTCs) is a pressing public health challenge, especially among the ageing population.^1^ The bidirectional relationship between depression and MLTCs worsens physical and mental health, reduces quality of life, increases mortality and raises healthcare costs.^2^ The global COVID-19 pandemic further complicated these challenges, with social distancing and ‘lockdowns’ (periods during which people were required to stay at home except for essential activities from May 2020 to 2021) restricting personal liberty. These measures, known as ‘shielding’, for individuals deemed ‘clinically extremely vulnerable’^3^ heightened concerns about health deterioration due to increased loneliness and social isolation. Studies indicate that loneliness and social isolation not only harm the mental and physical health of older adults with depression^4^ but also exacerbate depression for up to 12 years.^5^ This highlighted an urgent need during the COVID-19 pandemic for an effective remote intervention to reduce depressive symptoms, alleviate loneliness and potentially enhance physical health.

Behavioural activation (BA) is an evidence-based psychological treatment that alleviates depressive symptoms by recognising the link between physical inactivity and low mood, disrupting the cycle of depression and encouraging activities that boost positive reinforcement.^6^ BA is known to be effective in treating depression among older adults and addressing depressive symptoms associated with social isolation, making it particularly suitable for situations such as COVID-19 restrictions. Small trials have explored its effectiveness for socially isolated older individuals, with or without depression.^7 8^ For instance, Choi *et al* found that telephone-delivered BA improved social connectedness and reduced depression in 89 homebound older adults in the USA compared with friendly telephone visits (active control).^7^ Similarly, Pellas *et al* reported that a 4-week telephone-based BA intervention during COVID-19 significantly reduced depression symptoms in 41 participants in Sweden (between-group effect size Hedge’s g=0.85) compared with an attention-assessment control group.^8^ These findings indicate BA as a feasible, acceptable and promising intervention for treating depressive symptoms in isolated older adults.

Although the effectiveness of BA for older adults experiencing depression and isolation has been investigated, its cost-effectiveness for those with depression comorbid with MLTCs or living in isolation remains unexplored. However, evidence from related populations suggests BA may be cost-effective for this group. For example, Richards *et al* found that non-specialist-delivered BA reduced costs and improved quality-adjusted life year (QALY) outcomes compared with cognitive–behavioural therapy in 221 adults with depression (incremental cost −£343.24, 95% CI −857.62 to 171.13; incremental QALY 0.050, 95% CI −0.046 to 0.145).^9^ Similarly, Janssen *et al* reported BA was cost-effective compared with treatment as usual in 161 older adults with depression in Dutch primary care (incremental cost −€485, 95% CI −3861 to 2792; incremental QALY 0.026, 95% CI −0.0037 to 0.055).^10^

## Objective

The aim of this study was to assess the cost-utility of BA compared with usual care for older adults with depression and MLTCs during COVID-19 restrictions, following the Consolidated Health Economic Evaluation Reporting Standards 2022 guidelines (online supplemental appendix 1).

## Methods

### Trial design

The economic evaluation was embedded within the Behavioural Activation in Social IsoLation (BASIL+) trial, a two-arm, pragmatic, parallel-group, multicentre, randomised control trial (RCT) comparing remotely delivered BA with usual care for older adults experiencing depression and MLTCs during COVID-19 restrictions. Full details are available elsewhere.^11^ In brief, participants aged 65+ with two or more LTCs (defined by the Department of Health^12^) or considered clinically extremely vulnerable to COVID-19 according to the UK Health Security Agency, and experiencing low mood or depression (with scores ≥5 on the Patient Health Questionnaire (PHQ-9)^13^) were recruited via general practices across England and Wales (8 February 2021–28 Feb 2022). No participants were excluded due to having a specific combination of MLTCs that might limit their physical activity. Detailed inclusion/exclusion criteria are in online supplemental appendix 2. After eligibility assessment and consent, participants were randomised in a 1:1 ratio using blocked randomisation stratified by site. The intervention arm received BA and a self-help booklet, while control participants received the usual care with signposting to reputable self-help resources. All participants were followed up at 1, 3 and 12 months after randomisation. A study flowchart is in online supplemental appendix 3.

### Intervention

BA, a structured psychological intervention,^6^ was adapted for the BASIL+ trial to mitigate depression and loneliness during COVID-19 restrictions. Delivered remotely by trained BASIL support workers (BSWs), BA involved the collaboratively developing plans to reinstate or replace behaviours connecting participants to valued activities. Participants were offered up to eight (ideally weekly) sessions supported by the self-help BASIL+ BA booklet. Initial session lasted up to an hour, with subsequent ones around 30 minutes. The booklet provides practical strategies for adaption activities within pandemic restrictions.

### Cost measurements

The study considered the BA intervention costs and the wider service use costs. BA intervention costs, collected via a tailored questionnaire completed by the study team, included training (BSW manual and video recording for online self-learning, and staff time for online workshops) and delivery. Intervention delivery costs were obtained directly from the BSWs and their clinical supervisors and included time spent planning and delivering sessions, engaging in regular supervision sessions and material costs for booklet production. Total intervention costs, measured via a bottom-up costing approach,^14^ were allocated to each older adult receiving the intervention.

Service-use costs were measured using a tailored resource-use questionnaire completed by the participants at baseline, 1, 3 and 12 months postrandomisation, with varied recall periods of 1 month at baseline, and 1, 2 and 9 months at 1, 3 and 12 months follow-up, respectively. The data covered healthcare service utilisation (including both community-based and hospital-based services), private expenses and costs of informal care. Service use costs were calculated using a bottom-up approach,^14^ based on the quantity of collected resource use information multiplied by unit costs obtained from published sources: the National Cost Collection 2021/22,^15^ the Unit Costs of Health and Social Care report 2021 produced by the Personal Social Services Research Unit^16^ and the Prescription Cost Analysis—England 2021.^17^ Private service costs were valued at market prices, and informal care was valued using the national average. Key unit costs and assumptions are provided in online supplemental appendix 4. All costs were expressed in 2021 UK sterling, with no discounting applied due to the 12-month study timeframe.^18^

### Health outcome measurements

Health outcomes were assessed using QALYs, measured by the EQ-5D-3L^19^ instead of 5L, as the 3L is widely used and recommended by the National Institute for Health and Care Excellence (NICE) in its Guide to the Methods of Technology Appraisal,^20^ along-side the Short-Form Six-Dimension (SF-6D).^21^ Similar to the resource-use questionnaire, the EQ-5D-3L and SF-12v2 were collected at baseline, 1, 3 and 12 months postrandomisation. The EQ-5D-3L is a five-item generic preference-based measure of health-related quality of life (HRQoL) across five dimensions (mobility, self-care, usual activities, pain/discomfort and anxiety/depression), with severity levels ranging from 1 (best state) to 3 (worst state). The SF-6D, derived from the SF-12v2 questionnaire, is a 6-item HRQoL measure^21^ with four to six severity levels over six dimensions: physical functioning, role limitations, social functioning, pain, mental health and vitality. Individual-level responses of both instruments were used to estimate utilities based on UK adult population tariffs.^22 23^ A utility represents a health state ‘today’ and ranges from 1 (full health) to 0 (death) and less than 0 (worse than death). QALYs over 12 months were calculated by combining baseline and follow-up utilities using the ‘area under the curve’ approach.^14^

### Missing data

Two sample groups were considered in this study. The ‘complete case’ group refers to the participants with complete utility and cost data at all time points; while the ‘base case’ group refers to all the participants who had complete baseline assessments, even if utility and/or cost data were missing. Missing utility and cost data were imputed using multiple imputation via chained equations,^24^ performed by the trial arm at the level of individual items with 30 imputation sets, based on age, study site and baseline EQ-5D-3L utility and costs from NHS and personal social service (NHS/PSS) perspective.

### Cost-utility and sensitivity analyses

The primary analysis was a within-trial cost-utility analysis (CUA) based on an intention-to-treat approach, calculating an incremental cost-effectiveness ratio (ICER) based on the costs from the NHS/PSS perspective and the QALYs measured by EQ-5D-3L over 12 months.

To address uncertainty around the ICER estimate and potential imbalances in utility and cost at baseline, a multivariate multilevel model (MMLM) was used to adjust for clustering by study sites and control for age, utility and cost at baseline. The MMLM approach considers the distribution of the dependent variable and the correlation between cost and QALY outcomes.^25^ A non-parametric bootstrap method, suggested by Briggs and colleagues^26^ due to likely skewness in regression residuals, was performed with 5,000 iterations, which were deemed sufficient to generate robust SE estimates^26^ and is widely used in trial-based CUA for mental health conditions.^27^ Bootstrap and multiple imputation were combined by applying analysis models to each imputed dataset within each Bootstrap sample. Results were pooled across 30 imputed datasets using Rubin’s rules to combine estimates,^24^ yielding a total of 5,000 pooled bootstrapped results. The NICE recommended willingness-to-pay threshold (£20,000–£30 000 per QALY gained) was used to assess the cost-effectiveness.^16^ Bootstrapped results were presented on the cost-effectiveness plane (CE-plane), and a cost-effectiveness acceptability curve (CEAC) showed the probability of BA being cost-effective across various thresholds.^14^

A set of sensitivity analyses was conducted to test various assumptions and evaluate the robustness of the findings: a complete-case CUA, a CUA from a societal perspective (including private expenses and informal care costs) to capture wider economic impacts beyond the NHS/PSS perspective, a CUA using the SF-6D to assess the effect of the outcome measurement instrument and a CUA from the NHS/PSS perspective excluding training costs to examine the impact of providing the intervention over an extended period, thereby eliminating the need for continuous training. Additionally, as an ad-hoc analysis, a CUA that removed high-volume but plausible cases was conducted to test their impact on the primary analysis results.

All analyses were predefined in the health economics analysis plan, which is available on request from the corresponding author (HW), and performed using Stata V.16 (StataCorp, College Station, Texas, USA) and R V.4.2.3 (R Foundation for Statistical Computing, Vienna, Austria).

## Findings

### Baseline characteristics

Overall, 435 participants from 26 general practices were randomised (218 for BA and 217 for usual care). Among them, 281 (64.6%) participants had both EQ-5D-3L and resource use data (from the NHS and PSS perspective) available at all time points. The baseline characteristics of the 435 participants (base case) and 281 participants (complete case) are presented in table 1. Approximately one-third of older adults in both arms were male, and the average age was around 75 years. Differences in the EQ-5D-3L and SF-6D utility scores at baseline were very small across arms and samples (base case and complete case). Baseline characteristics were consistent across samples and aligned with the main statistical analysis.^11^ Details about missing data and patterns of missingness on resource use and utility are reported in online supplemental appendix 5.

**Table 1:** Baseline characteristics

Baseline characteristics	Base case (n=435)		Complete case (N=281)	
	BA (n=218)	Usual care (n=217)	BA (n=125)	Usual care (n=156)
Gender, n (%)				
Male	81 (37.2%)	84 (38.7%)	41 (32.8%)	60 (38.5%)
Age (years)				
Mean (SD)	74.7 (6.4)	75.7 (6.9)	74.5 (6.2)	75.3 (6.8)
Ethnicity, n (%)				
White	204 (93.6%)	203 (93.6%)	118 (94.4%)	147 (94.2%)
Non-white	14 (6.4%)	14 (6.4%)	7 (5.6%)	9 (5.8%)
Long-term condition, n (%)*				
Cardiovascular condition	144 (66.1%)	144 (66.4%)	83 (66.4%)	108 (68.8%)
Arthritis	98 (45.0%)	88 (40.6%)	58 (46.4%)	60 (38.2%)
Respiratory condition	60 (27.5%)	61 (28.1%)	36 (28.8%)	41 (26.1%)
Diabetes	77 (35.3%)	60 (27.7%)	45 (36.0%)	46 (29.3%)
Stroke	12 (5.5%)	16 (7.4%)	4 (3.2%)	13 (8.3%)
Chronic pain	44 (20.2%)	37 (17.1%)	28 (22.4%)	27 (17.2%)
Osteoporosis	19 (8.7%)	14 (6.5%)	11 (8.8%)	8 (5.1%)
Neurological condition	23 (10.6%)	16 (7.4%)	15 (12.0%)	12 (7.6%)
Cancer	26 (11.9$)	26 (12.0%)	14 (11.2%)	15 (9.6%)
Other	46 (21.1%)	51 (23.5%)	27 (21.6%)	38 (24.2%)
EQ-5D-3L utility score				
Mean (SD)	0.61 (0.27)	0.62 (0.28)	0.63 (0.26)	0.61 (0.28)
SF-6D utility score				
Mean (SD)	0.55 (0.26)	0.54 (0.26)	0.56 (0.26)	0.53 (0.25)
Baseline cost (£)				
Mean (SD)	487.78 (580.33)	579.68 (701.13)	497.12 (672.72)	575.54 (732.92)
Number of BA sessions				
Mean (SD)	5.17 (2.93)	–	6.50 (2.17)	–
Cost of BA intervention per participant (£)				
Training cost	18.69	–	18.69	–
Booklet cost	4.01		4.01	
Delivery cost (SD)	89.02 (55.51)	–	85 (47.98)	–
Supervision cost (SD)	15.26 (13.63)		18.55 (14.07)	

* Conditions are not mutually exclusive, so percentages are not expected to sum to 100.

BAbehavioural activationSF-6DShort-Form Six-Dimension

### Costs

On average, participants received 5.17 BA sessions (table 1), and the average intervention cost for BA was £127.00 (£18.69 for training and £108.31 for intervention delivery). The key cost driver for training costs was trainer’s time (£14.18), while the main cost drivers for intervention delivery costs were preparation (£30.20) and delivery time of the intervention (£58.82). Online supplemental appendix 6 provides detailed information on the cost breakdown. No intervention costs were assumed for participants in the usual care arm.

Online supplemental appendix 7 presents a summary of resource use over 12 months, and table 2 breaks down costs by perspective, service type, trial arm and before and after imputation. Average costs for service use were similar between the two arms, with older adults in the BA arm incurring slightly lower average service use costs compared with those in the usual care arm, except for community services and informal care. Overall, total service costs to NHS and PSS providers (rounded to the nearest pound for the base case) were slightly lower for BA (£1,403.54, 95% CI: 1,258.87 to 1,548.20) compared with usual care (£1,548.57, 95% CI: 1,384.17 to 1712.96). This trend is also observed for costs to society, both in the complete case and the base case.

**Table 2:** Average costs of service use in 12 months by the trial arm (costs reported in 2021 £)

	Base case		Complete case	
	BA (n=218)£ (95% CI)	Usual care (n=217)£ (95% CI)	BA (n=94)£ (95% CI)	Usual care (n=96)£ (95% CI)
NHS and PSS	1403.54 (1258.87, 1548.20)	1548.57 (1384.17, 1712.96)	1429.79 (1239.93, 1619.65)	1582.94 (1380.37, 1785.51)
Community-based services	250.71 (221.11, 280.31)	281.26 (231.61, 330.91)	249.08 (211.47, 286.71)	289.67 (226.02, 353.32)
GP	191.86 (166.70, 217.02)	231.06 (184.50, 277.63)	210.47 (138.33, 282.61)	235.26 (174.35, 296.17)
Community services	58.85 (42.54, 75.16)	50.20 (33.01, 67.38)	61.46 (41.05, 81.87)	55.73 (34.34, 77.13)
Hospital-based services	543.74 (423.38, 664.10)	624.01 (496.66, 751.37)	565.71 (422.12, 709.30)	644.81 (488.71, 800.91)
Medications	609.09 (555.34, 662.84)	643.29 (587.64, 698.95)	614.99 (543.20, 686.78)	647.14 (580.19, 714.10)
Private expenses	25.36 (5.86, 44.87)	33.90 (12.65, 55.15)	34.45 (3.77, 65.13)	42.30 (13.50, 71.11)
Informal care	191.40 (138.05, 244.76)	171.19 (126.07, 216.31)	180.74 (119.11, 242.37)	177.81 (125.07, 230.55)
Total costs	1620.30 (1460.86, 1779.74)	1753.66 (1573.34, 1933.98)	1644.98 (1432.33, 1856.62)	1803.05 (1581.29, 2024.82)

BAbehavioural activationGPgeneral practicePSSpersonal social service

### Health outcomes

Table 3 provides the mean utility scores measured by EQ-5D-3L and SF-6D across both arms at each time point for both the complete and base cases (after imputation), while illustrative plots of these scores are presented in online supplemental appendix 8. As shown, in both arms, there was a small increase (0.01–0.04) in EQ-5D-3L utility scores from baseline to 3 months, suggesting a slight improvement in quality of life. However, at 12 months, EQ-5D-3L scores declined in both arms, with a more pronounced reduction observed in the BA arm than in the usual care arm. Conversely, a small and consistent increase in utility scores was observed from baseline to 12 months when measured by the SF-6D. Furthermore, BA resulted in a similar mean QALY improvement compared with usual care, whether measured by EQ-5D-3L (0.63 QALYs) or SF-6D (0.58 QALYs). This consistency was observed in both the base and complete case analyses. Further details regarding the responses of EQ-5D-3L and SF-6D in each domain can be found in online supplemental appendix 9 and 10, respectively.

**Table 3:** Average EQ-5D-3L and SF-6D utility scores by trial arm

EQ-5D-3L	Base case		Complete case	
	BA (n=218)Mean (95% CI)	Usual care (n=217)Mean (95% CI)	BA (n=125)Mean (95% CI)	Usual care (n=157)Mean (95% CI)
Utility score				
Baseline	0.61 (0.57, 0.64)	0.62 (0.58, 0.66)	0.63 (0.58, 0.68)	0.61 (0.57, 0.66)
1 month	0.64 (0.60, 0.68)	0.62 (0.58, 0.66)	0.65 (0.60, 0.70)	0.62 (0.57, 0.66)
3 months	0.64 (0.60, 0.68)	0.64 (0.60, 0.68)	0.64 (0.59, 0.69)	0.65 (0.60, 0.69)
12 months	0.58 (0.53, 0.64)	0.60 (0.56, 0.65)	0.57 (0.52, 0.63)	0.60 (0.56, 0.65)
Total QALYs	0.63 (0.58, 0.68)	0.63 (0.59, 0.66)	0.62 (0.57, 0.66)	0.62 (0.59, 0.66)
**SF-6D**	**Base case**		**Complete case**	
	**BA (n=218)Mean (95% CI**)	**Usual care (n=217)Mean (95% CI**)	**BA (n=120)Mean (95% CI**)	**Usual care (n=153)Mean (95% CI**)
Utility score				
Baseline	0.55 (0.52, 0.59)	0.54 (0.50, 0.57)	0.56 (0.51, 0.61)	0.53 (0.49, 0.57)
1 month	0.56 (0.52, 0.60)	0.57 (0.53, 0.61)	0.56 (0.51, 0.60)	0.57 (0.53, 0.61)
3 months	0.57 (0.53, 0.61)	0.59 (0.55,0.62)	0.58 (0.54, 0.63)	0.58 (0.54, 0.62)
12 months	0.60 (0.56, 0.65)	0.58 (0.54, 0.62)	0.60 (0.56, 0.65)	0.57 (0.53, 0.61)
Total QALYs	0.58 (0.54, 0.62)	0.58 (0.54, 0.61)	0.58 (0.54, 0.62)	0.58 (0.54, 0.61)

BAbehavioural activationQALYquality-adjusted life yearSF-6DShort-Form Six-Dimension

### Primary CUA

**Figure 1:**
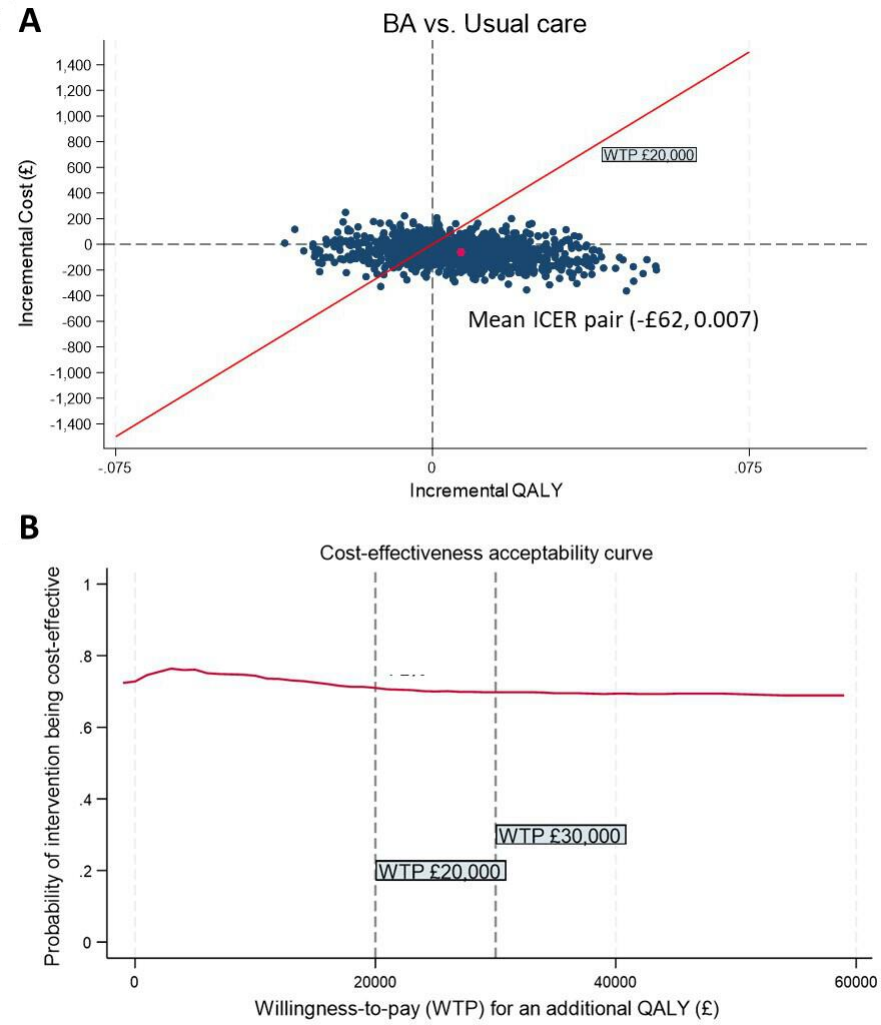
Cost-effectiveness plane and CEAC of the primary analysis (QALY measured by EQ-5D-3L, costs measured from an NHS/PSS perspective). BA, behavioural activation; CEAC, cost-effectiveness acceptability curve; ICER, incremental cost-effectiveness ratio; PSS, personal social service; QALY, quality-adjusted life year.

The primary analysis focused on determining the ICER based on the base case. After considering uncertainty and adjusting for imbalanced utility scores and healthcare costs at baseline, the results indicate that, on average, at-risk socially isolated older adults receiving BA incurred £62.34 (95% CI −£120.44 to £239.70) less costs and produced 0.007 (95% CI −0.036 to 0.022) more QALY improvements compared with those receiving usual care. Figure 1A shows that approximately three-quarters of the bootstrapped estimates fall below the £20,000 threshold line, residing mainly in the south-east quadrant. This suggests that, although the observed cost reduction and QALY improvement were negligible and not statistically significant, BA was still highly likely to be the preferred option compared with usual care. This finding is further supported by the CEAC (figure 1B), which indicates that the estimated probability of BA being cost-effective is 0.71 when decision makers are willing to pay £20,000 for one QALY gained.

### Sensitivity analyses

**Figure 2:**
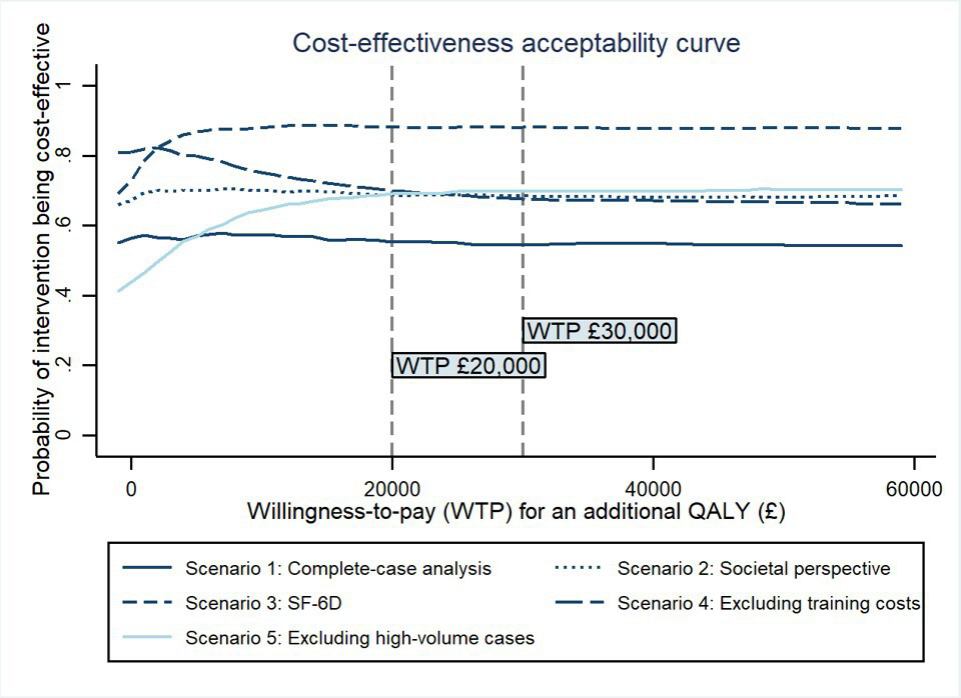
Cost-effectiveness acceptability curves (CEACs) of sensitivity analyses. QALY, quality-adjusted life year; SF-6D, Short-Form Six-Dimension.

Results of a set of sensitivity analyses are illustrated in figure 2, with detailed information provided in online supplemental appendix 11 and cost-effectiveness plane plots in online supplemental appendix 12. At a willingness-to-pay threshold of £20,000 per QALY gained, the probability of the BA intervention being cost-effective was consistent with the base case when costs were measured from a societal perspective (0.686 in figure 2, scenario 2), when QALYs were measured using the SF-6D (0.882 in figure 2, scenario 3), when training costs were excluded as a one-off cost based on an NHS/PSS perspective (0.700 in figure 2, scenario 4), and when high-volume but plausible cases were removed (0.690 in figure 2, scenario 5, with further details provided in the online supplemental file 1 and online supplemental appendices 11 and 12. Although the acceptance probability was slightly lower in the complete case analysis (0.55 in figure 2, scenario 1), all the sensitivity analyses indicated that BA dominated usual care, especially when QALYs were measured using the SF-6D. This affirmed that BA is likely to be the preferred option compared with usual care.

## Discussion

### Summary of findings and comparison to literature

To the best of our knowledge, this is the first economic evaluation of BA in socially isolated older adults with both depression and MLTCs. Our results indicate that, compared with usual care, BA did not lead to an increase in the service use costs to the NHS nor to society, while maintaining similar QALY improvements over 12 months. The probability of BA being cost-effective is 0.71, suggesting that BA is likely to be a preferred option for the NHS compared with usual care, although with some level of uncertainty. This is evident in both the primary and sensitivity analyses. Our findings also align with previous cost-effectiveness studies in BA for treating depression or subthreshold depression (without MLTCs),^9 10 28^ affirming its position as a preferred option.

### Study implications

After adjusting for imbalanced baseline characteristics and considering uncertainty, a small but non-significant cost reduction was observed for BA compared with usual care across NHS/PSS and societal perspectives. This cost-saving result is primarily attributed to reduced access to health services, which may be influenced by the BA intervention or potentially by noises, such as imbalanced COVID-19 infection rates in the control arm. The exact mechanisms behind these findings remain unclear and warrant further investigation. It is also important to note that the study was conducted during the COVID-19 pandemic, and thus, the findings may be most applicable to similar contexts characterised by pandemic-related restrictions, although the conditions in our study population are not specific to a COVID-19 pandemic setting. The study team is currently exploring the same remote BA intervention in non-pandemic settings without lockdown measures (Multimorbidity in Older Adults with Depression Study trial).^29^ The forthcoming results will help determine whether the findings from this study can be generalised to typical, non-pandemic environments. Despite the modest nature of these results, the finding still holds relevance for commissioners tasked with scaling interventions to a national level, especially during public health crises such as the COVID-19 pandemic. The potential cost saving to the NHS during such nationwide pandemic-induced isolation in the UK is estimated to exceed £75 million. This estimation is derived from the fact that currently there are over 12 million adults aged 65 and over in the UK^30^; depression affects around 22% of older men and 28% of older women,^31^ and approximately 40% of depressive older adults have MLTCs.^32^ However, our finding needs to be interpreted with caution for three reasons. First, the cost reduction was not statistically significant. Second, a comprehensive long-term analysis is necessary to ensure the sustained effectiveness of BA over an extended long period beyond the 12-month timeframe, and to verify the persistence of the associated cost savings. Third, the intervention costs in this study are relatively low compared with other UK-based BA studies, where BA intervention costs ranged from £247 per participant in 2011^33^ to £974.81 per participant in 2016.^9^ This cost difference may be due to several factors, including remote delivery of intervention training using a blended approach of direct online training and self-directed learning, and the delivery of BA by non-specialists such as nurses and social prescribers instead of clinical psychologists or psychiatrists, which typically incur higher costs. It is also worth noting that two high-volume cases—one receiving 120 community-based services and another having 336 carer sessions over 3 months—in the usual care arm at baseline were observed and retained without any alterations because they were deemed plausible. However, the impact of such high-value cases at the baseline is considered to be small, as the cost-effectiveness outcomes of excluding these two high-volume cases remain consistent to the primary analysis outcomes. The detailed results can be found in online supplemental appendices 11 and 12.

The difference in QALYs measured by EQ-5D-3L between the two groups over 12 months was relatively small (0.007), aligning with findings from studies on older adults with depression by Pizzi (0.0076)^27^ and Bosanquet (0.019).^26^ The improvement in HRQoL might be considered larger if the timeliness and convenience of BA during COVID-19 could be quantitatively captured, especially considering the qualitative evidence that older adults valued and appreciated BA. Furthermore, although BA does not appear to improve QALYs in the long run (12 months) compared with usual care, short-term improvements by BA were observed. BA increased participants’ utility scores over 1 month, and the impact endured until the 3-month follow-up. Our study also demonstrated that the choice of utility instrument can affect utility and QALY measurements. Utility scores were different when measured by different instruments, with utility scores tending to be always lower when measured by SF-6D compared with EQ-5D-3L. Two further differences were also observed. First, a small but consistent increase in utility scores was observed from baseline to 12 months when measured by SF-6D, while when measured by EQ-5D-3L, a steep increase in utility scores was observed up to 3 months followed by a decline at 12 months. Second, the incremental QALYs were larger for SF-6D than for EQ-5D-3L. One possible explanation may be that the SF-6D is more sensitive to change than the EQ-5D-3L, as the SF-6D contains more dimensions relevant to the intervention and to the mental health of older adults (eg, social functioning and vitality). The mechanism of such differences remains unknown and requires further investigation. For a future study, using the EQ-5D-5L, which offers more response levels for potentially greater precision and sensitivity in measuring QALY, as suggested by Pizzi *et al*,^28^ may be beneficial. Also, mental health-specific utility instruments could be used for greater sensitivity to changes in depression symptoms.

### Strengths and limitations

This study is the first to attempt to assess the cost-effectiveness of BA for socially isolated older adults with depression and MLTCs. The data were collected from a fully powered RCT, offering more robust estimates compared with small-scale studies. The economic evaluation adopts a multi-perspective approach with different costing considerations, ensuring the study’s robustness and making the results valuable to a wide range of stakeholders, including health policy makers and healthcare providers. Furthermore, the study results have implications for future pandemics and for policy decisions about managing depression and MLTCs in socially isolated adults.

There were, however, some limitations. A simplified participant self-report health resource use questionnaire was designed to reduce the response burden of participants completing the study during the COVID-19 restrictions. This makes it challenging to differentiate cost differences between the two arms and could potentially introduce bias to our results, as a few strong assumptions and national averages were necessary for the costing. For example, medication costs were estimated based on the number of medications participants reported taking and national average medication cost, rather than collecting specific medication details and their respective costs. The decision not to collect this more detailed information was in part taken to reduce participant burden and was guided by the study’s patient and public involvement advisory group. We acknowledge that this approach may have led to an underestimation of true medication costs, particularly given the study population’s age and their MLTCs. However, since the assumptions applied to both arms are the same and sensitivity analyses demonstrated consistent results in line with our primary analysis results, it is expected that the impact on our results is limited. For future studies on social isolation, a more detailed health resource use questionnaire is recommended to fully capture cost differences for more robust cost estimations. Furthermore, in this study, ‘Did not attend’ or cancelled sessions of the BA intervention were not included in the costing, which may have led to an underestimation of the intervention’s true cost. Future studies would benefit from incorporating these missed or cancelled sessions into the costing analysis.

A non-negligible amount of missing data for the primary analysis may introduce bias to the research outcomes and limit the precision of the conclusions. While the presence of these missing data does introduce additional uncertainty with regard to the substantive conclusion, the apparent insensitivity of the results to the missing-at-random assumption provides reassurance.

Study results are confined to cost-effectiveness of BA over a 12-month time horizon. Although outside the scope of the current study, considering the long-term impact of isolation on individuals with depression and MLTCs using a model-based economic evaluation would be desirable in future research to allow long-term cost-effectiveness to be measured.

### Clinical implications

The implementation of remotely delivered BA for older adults with depression and MLTCs during COVID-19 restrictions showed no increase in costs while maintaining comparable QALY improvements compared with usual care during COVID-19. This suggests that remotely delivered BA is a promising intervention for managing depression in isolated older adults with MLTCs amidst public health challenges, including future pandemics.

## supplementary material

10.1136/bmjment-2024-301270 online supplemental file 1

## Data Availability

Data are available upon reasonable request.
