# Supplementary material for: Cost-utility of behavioural activation for mitigating psychological impacts of COVID-19 on socially isolated older adults with depression and multiple long-term conditions compared with usual care: results from a pragmatic randomised controlled trial
Source: BMJ Ment Health. 2025 Jan 19;28(1):e301270. doi: 10.1136/bmjment-2024-301270 (PMC11751961; doi:10.1136/bmjment-2024-301270)

Appendix

Appendix 1: CHEERS 2022 Checklist

	Item	Guidance for Reporting	Reported in section
**TITLE**			
Title interventions	1	Identify the study as an economic evaluation and specify the being compared.	Page 1
**ABSTRACT**			
Abstract	2	Provide a structured summary that highlights context, key methods, results and alternative analyses.	Page 1
**INTRODUCTION**			
Background and objectives	3	Give the context for the study, the study question and its practical relevance for decision making in policy or practice.	Page 3-4
**METHODS**			
Health economic analysis plan	4	Indicate whether a health economic analysis plan was developed and where available.	Page 7
Study population	5	Describe characteristics of the study population (such as age range, demographics, socioeconomic, or clinical characteristics).	Page 4, Section 1
Setting and location	6	Provide relevant contextual information that may influence findings.	Page 4, Section 1
Comparators	7	Describe the interventions or strategies being compared and why chosen.	Page 4, Section 1
Perspective	8	State the perspective(s) adopted by the study and why chosen.	Page 6
Time horizon	9	State the time horizon for the study and why appropriate.	Page 5 and 6
Discount rate	10	Report the discount rate(s) and reason chosen.	Page 5
Selection of outcomes	11	Describe what outcomes were used as the measure(s) of benefit(s) and harm(s).	Page 6
Measurement of outcomes	12	Describe how outcomes used to capture benefit(s) and harm(s) were measured.	Page 6
Valuation of outcomes	13	Describe the population and methods used to measure and value outcomes.	Page 6
Measurement and valuation of resources and costs	14	Describe how costs were valued.	Page 5
Currency, price date, and conversion	15	Report the dates of the estimated resource quantities and unit costs, plus the currency and year of conversion.	Page 5
Rationale and description of model	16	If modelling is used, describe in detail and why used. Report if the model is publicly available and where it can be accessed.	N/A
Analytics and assumptions	17	Describe any methods for analysing or statistically transforming data, any extrapolation methods, and approaches for validating any model used.	Page 6-7
Characterizing heterogeneity	18	Describe any methods used for estimating how the results of the study vary for sub-groups.	Page 7
Characterizing distributional effects	19	Describe how impacts are distributed across different individuals or adjustments made to reflect priority populations.	N/A
Characterizing uncertainty	20	Describe methods to characterize any sources of uncertainty in the analysis.	Page 6
Approach to engagement with patients and others affected by the study	21	Describe any approaches to engage patients or service recipients, the general public, communities, or stakeholders (e.g., clinicians or payers) in the design of the study.	Page 16
**RESULTS**			
Study parameters	22	Report all analytic inputs (e.g., values, ranges, references) including uncertainty or distributional assumptions.	N/A
Summary of main results	23	Report the mean values for the main categories of costs and outcomes of interest and summarise them in the most appropriate overall measure.	Page 7-10
Effect of uncertainty	24	Describe how uncertainty about analytic judgments, inputs, or projections affect findings. Report the effect of choice of discount rate and time horizon, if applicable.	Page 10 and 12
Effect of engagement with patients and others affected by the study	25	Report on any difference patient/service recipient, general public, community, or stakeholder involvement made to the approach or findings of the study	N/A
**DISCUSSION**			

Study findings, limitations, generalizability, and current knowledge	26	Report key findings, limitations, ethical or equity considerations not captured, and how these could impact patients, policy, or practice.	Page 12-15
**OTHER RELEVANT INFORMATION**			
Source of funding	27	Describe how the study was funded and any role of the funder in the identification, design, conduct, and reporting of the analysis	Page 16
Conflicts of interest	28	Report authors conflicts of interest according to journal or International Committee of Medical Journal Editors requirements.	Page 16

Husereau D, Drummond M, Augustovski F, de Bekker-Grob E, Briggs AH, Carswell C, Caulley L, Chaiyakunapruk N, Greenberg D, Loder E, Mauskopf J, Mullins CD, Petrou S, Pwu RF, Staniszewska S; CHEERS 2022 ISPOR Good Research Practices Task Force. Consolidated Health Economic Evaluation Reporting Standards 2022 (CHEERS 2022) Statement: Updated Reporting Guidance for Health Economic Evaluations. BMJ. 2022;376:e067975.
The checklist is Open Access distributed in accordance with the terms of the Creative Commons Attribution (CC BY 4.0) license, which permits others to distribute, remix, adapt and build upon this work, for commercial use, provided the original work is properly cited. See: http://creativecommons.org/licenses/by/4.0/.

Appendix 2: Inclusion and exclusion criteria

Inclusion criteria

- Older adults (65 years or over)
- Two or more long-term conditions (LTCs) or a condition that may suggest they are within a ‘clinically extremely vulnerable’ group in relation to COVID-19
- A score of ≥5 at screening on the Patient Health Questionnaire (PHQ9)

Exclusion criteria

- Cognitive impairment
- Bipolar disorder/psychosis/psychotic symptoms
- Alcohol or drug dependence
- In the palliative phase of illness
- Have active suicidal ideation
- Currently receiving psychological therapy
- Unable to speak or understand English

Older adults will not be excluded on the basis of living in residential/care homes

Appendix 3: Trial flowchart

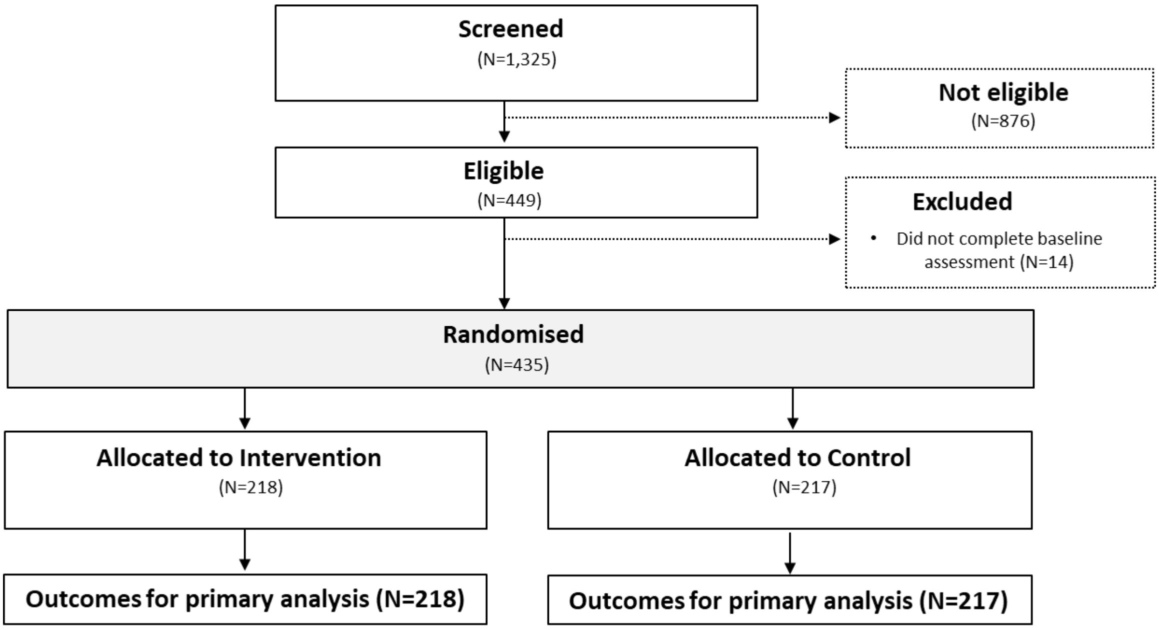

Appendix 4: Unit costs

Training and intervention

Item	Unit cost (£)	source
BASIL support worker	£19/hour	PSSRU 2021/22 (chapter 2.5)
Grade 6 research fellow	£21.00/hour	https://www.york.ac.uk/admin/hr/pay-and-grading/pay-scales/grades-1-8/
Grade 7 research fellow	£25.06/hour	https://www.york.ac.uk/admin/hr/pay-and-grading/pay-scales/grades-1-8/
Professor	£38.14/hour	https://www.york.ac.uk/admin/hr/pay-and-grading/pay-scales/professors/

Community-based services

Item	Unit cost (£)	source
General practitioner (GP)		
Face to face (at clinic / surgery)	£34.2	PSSRU 2021/22 (chapter 10.3b)
Via telephone	£37.7	PSSRU 2021/22 (chapter 10.4)
Via video	£37.7	PSSRU 2021/22 (chapter 10.4)
Home visit*	£44.12	PSSRU 2021/22 (chapter 10.3b)
Community service**	£23.0	PSSRU 2021/22 (chapter 11.1)

*Including the average travel time of 12 minutes per visit (PSSRU 2015).

**Assume the duration of visit is 30 minutes.

Hospital-based services

Item	Unit cost (£)	Source
Accident and Emergency	£134	PSSRU 2021/22 (chapter 7.1)
Outpatient visit	£137	PSSRU 2021/22 (chapter 7.1)
Video consultation	£137	Assume to be the same as outpatient visit
Inpatient stay	£4,754	PSSRU 2021/22 (chapter 7.1)

Medications

Item	Unit cost (£)	Source
Medication*	£8.5	PCA 2021 national average

*Assume one pack per month throughout the observation period.

Private expenses / additional support

Item	Unit cost (£)	Source
Cleaner*	£15	See below**
Carer*	£27	PSSRU 2021/22 (chapter 11.5)
Private dentist	£133	PSSRU 2021/22 (chapter 10.6)
Chiropody***	£50	https://www.painfreefeet.co.uk/chiropody-cost/

* Assume 1 hour per session.

**https://cleaning-express.com/how-much-does-a-cleaner-cost/#:~:text=Weekly%20cleaner%20cost,is%20%C2%A315%20per%20hour

***Assume 30 mins per session.

Informal care cost

Item	Unit cost (£)	Source
Average cost per day in the UK	£5.36	Pinedo-Villanueva, R., Westbury, L.D., Syddall, H.E. et al. Health Care Costs Associated With Muscle Weakness: A UK Population-Based Estimate. Calcif Tissue Int 104, 137–144 (2019). https://doi.org/10.1007/s00223-018-0478-1

* Assume the informal care costs is the same as the costs to the older adults with muscle weakness in the UK (£4.75 in 2015). The costs are inflated to 2021 based on the NHS Cost Inflation Index (NHSCII) of PSSRU.

Appendix 5: Missing data and patterns

	Baseline n (%)		1-month n (%)		3-month n (%)		12-month n (%)		Complete case n (%)	
**Total**	**BA n=218**	**Usual care n=217**	**BA n=218**	**Usual care n=217**	**BA n=218**	**Usual care n=217**	**BA n=218**	**Usual care n=217**	**BA n=218**	**Usual care n=217**
**Cost data**										
Resource use data from the NHS/PSS perspective	218 (100.0%)	217 (100.0%)	164 (75.2%)	187 (86.2%)	161 (73.9%)	190 (87.6%)	140 (64.2%)	169 (77.9%)	125 (57.3%)	156 (71.9%)
Resource use data from the societal perspective	218 (100.0%)	217 (100.0%)	164 (75.2%)	187 (86.2%)	161 (73.9%)	190 (87.6%)	148 (64.2%)	169 (77.9%)	125 (57.3%)	156 (71.9%)
**Health outcome data**										
EQ-5D-3L	218 (100.0%)	217 (100.0%)	164 (75.2%)	187 (86.2%)	161 (73.9%)	190 (87.6%)	140 (64.2%)	169 (77.9%)	125 (57.3%)	156 (71.9%)
SF-6D	217 (99.5%)	217 (100.0%)	164 (75.2%)	188 (86.6%)	161 (73.9%)	188 (86.6%)	137 (62.8%)	167 (77.0%)	122 (56.0%)	151 (69.6%)
**Economic evaluation**										
EQ-5D-3L and costs (NHS/PSS perspective)	218 (100.0%)	217 (100.0%)	164 (75.2%)	187 (86.2%)	161 (73.9%)	190 (87.6%)	140 (64.2%)	169 (77.9%)	125 (57.3%)	156 (71.9%)
EQ-5D-3L and costs (societal perspective)	218 (100.0%)	217 (100.0%)	164 (75.2%)	187 (86.2%)	161 (73.9%)	190 (87.6%)	140 (64.2%)	169 (77.9%)	125 (57.3%)	156 (71.9%)
SF-6D and costs (NHS/PSS perspective)	217 (99.5%)	217 (100.0%)	164 (75.2%)	186 (85.7%)	161 (73.9%)	188 (86.6%)	137 (62.8%)	167 (77.0%)	122 (56.0%)	151 (69.6%)

Missing Data plot

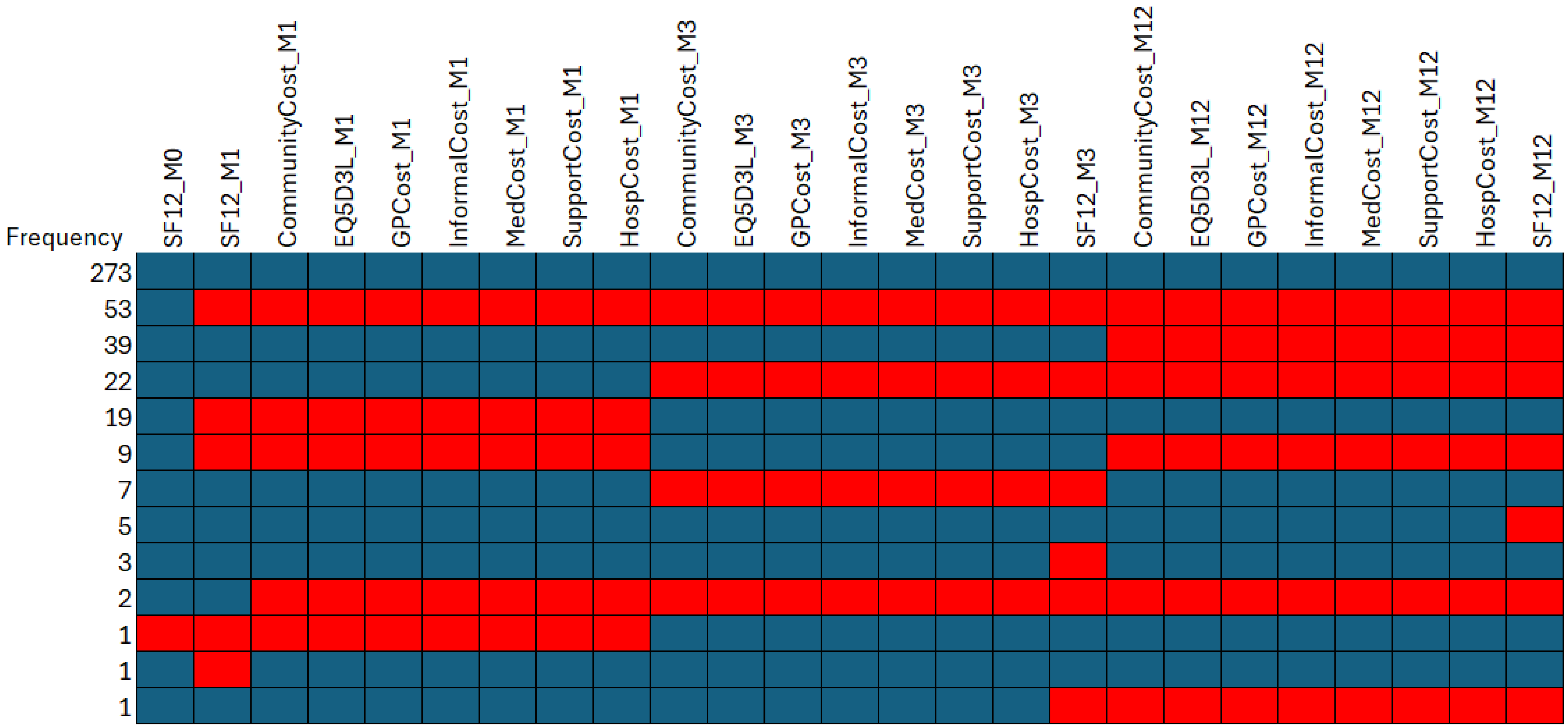

Appendix 6: Training and intervention delivery costs for the trial arm

	Total cost (£)	Cost per participant (£)[5]
**Online training costs**		
Manual	£835.56	£3.83
Print	£486.20	£2.23
Folder & jiffy bags	£129.36	£0.59
Postage	£220.00	£1.01
Video recording for online self-learning[1]	£147.00	£0.67
Trainers' staff time	£3,091.95	£14.18
Preparation	£512.10	£2.35
Training	£2,580.85	£11.84
**Total**	**£4,074.51**	**£18.69**
**Intervention delivery costs**		
Booklet	£874.18	£4.01
Printing and folders	£492.68	£2.26
Postage	£381.50	£1.75
Intervention (staff time)	£19,406.36	£89.02
Intervention session[2]	£12,822.76	£58.82
Administration / preparation[3]	£6,583.60	£30.20
Supervision[4]	£3,331.04	£15.28
**Total**	**£23,611.58**	**£108.31**
**Total (training and intervention delivery costs)**	**£27,686.09**	**£127.0**

[1] Two Grade 6 research fellows spent a total of 7 hours recording videos for online self-learning.
[2] The average total intervention session time, delivered by band 2-6 BASIL support workers, was 3.10 hours.
[3] The average total administration time, delivered by band 2-6 BASIL support workers, was 1.59 hours.
[4] The average total supervision time, delivered by two professors and one grade 7 research fellow, was 0.80 hours.
[5] Costs per participant were derived by dividing the costs by the 218 participants in the BA arm.

Appendix 7: Average service use by trial arm (BA, n=218; Usual care, n=217)

	Unit	Baseline		1 month		3 months		12 months	
		BA, Mean, median (min-max)	Usual care, Mean, median (min-max)	BA, Mean, median (min-max)	Usual care, Mean, median (min-max)	BA, Mean, median (min-max)	Usual care, Mean, median (min-max)	BA, Mean, median (min-max)	Usual care, Mean, median (min-max)
NHS and PSS									
General practice (GP)									
Face to face	Appointment	0.89, 0.00 (0-15)	0.99, 0.00 (0-30)	0.31, 0.00 (0-4)	0.36, 0.00 (0-4)	0.48, 0.00 (0-7)	0.48, 0.00 (0-10)	1.52, 1.00 (0-10)	1.35, 1.00 (0-12)
Via telephone	Call	1.37, 1.00 (0-12)	1.52, 1.00 (0-25)	0.61, 0.00 (0-6)	0.67, 0.00 (0-10)	0.75, 0.00 (0-6)	0.77, 0.00 (0-6)	1.66, 1.00 (0-20)	2.80, 1.00 (0-100)
Via video	Call	0.00, 0.00 (0-12)	0.01, 0.00 (0-1)	0.00, 0.00 (0-0)	0.00, 0.00 (0-0)	0.01, 0.00 (0-1)	0.00, 0.00 (0-0)	0.00, 0.00 (0-0)	0.01, 0.00 (0-1)
Home visit	Visit	0.04, 0.00 (0-6)	0.07, 0.00 (0-6)	0.00, 0.00 (0-0)	0.01, 0.00 (0-1)	0.03, 0.00 (0-2)	0.00, 0.00 (0-0)	0.06, 0.00 (0-4)	0.05, 0.00 (0-3)
Hospital-based services									
Outpatient visit	Attendance	0.86, 0.00 (0-7)	1.15, 0.00 (0-28)	0.41, 0.00 (0-8)	0.58, 0.00 (0-8)	0.84, 0.00 (0-20)	0.83, 0.00 (0-20)	1.50, 1.00 (0-12)	1.89, 0.00 (0-30)
Video consultation	Call	0.04, 0.00 (0-2)	0.02, 0.00 (0-2)	0.04, 0.00 (0-4)	0.03, 0.00 (0-3)	0.05, 0.00 (0-3)	0.02, 0.00 (0-1)	0.01, 0.00 (0-1)	0.02, 0.00 (0-4)
Inpatient admission	Day	0.10, 0.00 (0-6)	0.13, 0.00 (0-3)	0.01, 0.00 (0-1)	0.02, 0.00 (0-2)	0.07, 0.00 (0-3)	0.02, 0.00 (0-1)	0.09, 0.00 (0-3)	0.15, 0.00 (0-5)
Emergency services	Visit	0.09, 0.00 (0-3)	0.13, 0.00 (0-6)	0.01, 0.00 (0-1)	0.03, 0.00 (0-1)	0.04, 0.00 (0-1)	0.04, 0.00 (0-1)	0.20, 0.00 (0-4)	0.15, 0.00 (0-6)
Community services	Appointment	1.00, 0.00 (0-40)	1.66, 0.00 (0-120)	0.24, 0.00 (0-4)	0.42, 0.00 (0-20)	0.72, 0.00 (0-25)	0.54, 0.00 (0-20)	1.84, 0.00 (0-25)	1.34, 0.00 (0-42)
Medication	Type	6.41, 6.00 (0-25)	6.25, 6.00 (0-20)	5.97, 5.00 (0-28)	5.95, 5.00 (0-22)	5.83, 5.00 (0-20)	6.12, 5.00 (0-20)	6.06, 5.00 (0-28)	6.50, 5.00 (0-32)
Private expanses									
Cleaner*	Session	0.59, 0.00 (0-38)	0.44, 0.00 (0-36)	0.06, 0.00 (0-6)	0.04, 0.00 (0-4)	0.04, 0.00 (0-8)	0.18, 0.00 (0-16)	0.80, 0.00 (0-75)	0.17, 0.00 (0-24)
Carer*	Session	1.10, 0.00 (0-90)	2.79, 0.00 (0-336)	-	0.17, 0.00 (0-23)	-	0.64, 0.00 (0-56)	0.35, 0.00 (0-75)	0.51, 0.00 (0-84)
Private dentist	Appointment	-	0.00, 0.00 (0-1)	-	-	0.01, 0.00 (0-2)	-	-	0.02, 0.00 (0-2)
Chiropody	Appointment	-	0.10, 0.00 (0-21)	-	0.04, 0.00 (0-7)	0.07, 0.00 (0-8)	0.06, 0.00 (0-13)	0.00, 0.00 (0-1)	0.01, 0.00 (0-3)
Productivity costs									
Informal care	Day	14.06, 0.00 (0-90)	13.15, 0.00 (0-90)	2.91, 0.00 (0-30)	4.22, 0.00 (0-30)	7.09, 0.00 (0-61)	7.55, 0.00 (0-61)	24.97, 0.00 (0-183)	21.61, 0.00 (0-183)

*Assume one hour per session

Appendix 8: Mean EQ-5D-3L and SF-6D utility scores by trial arm by and analysis group (base case and complete case) across time points

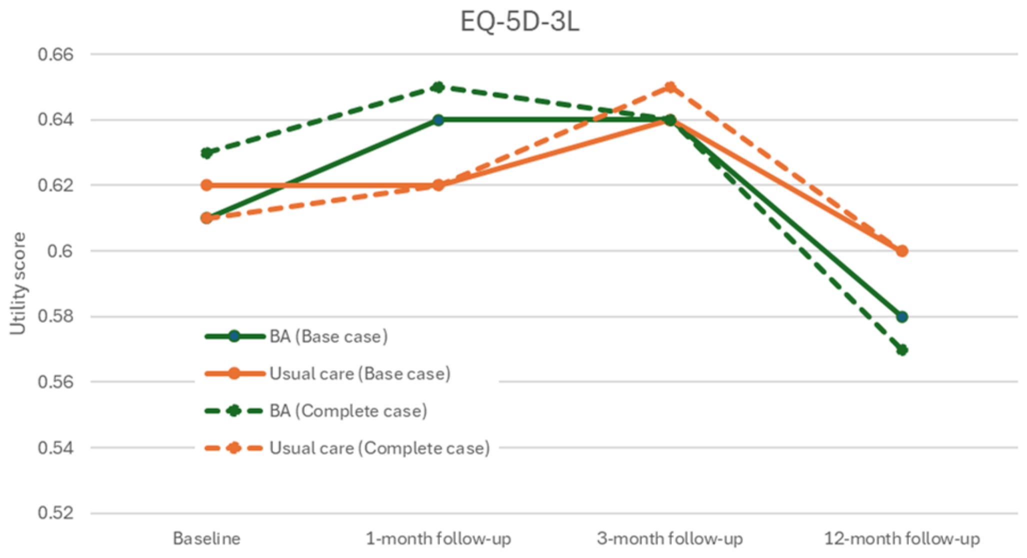

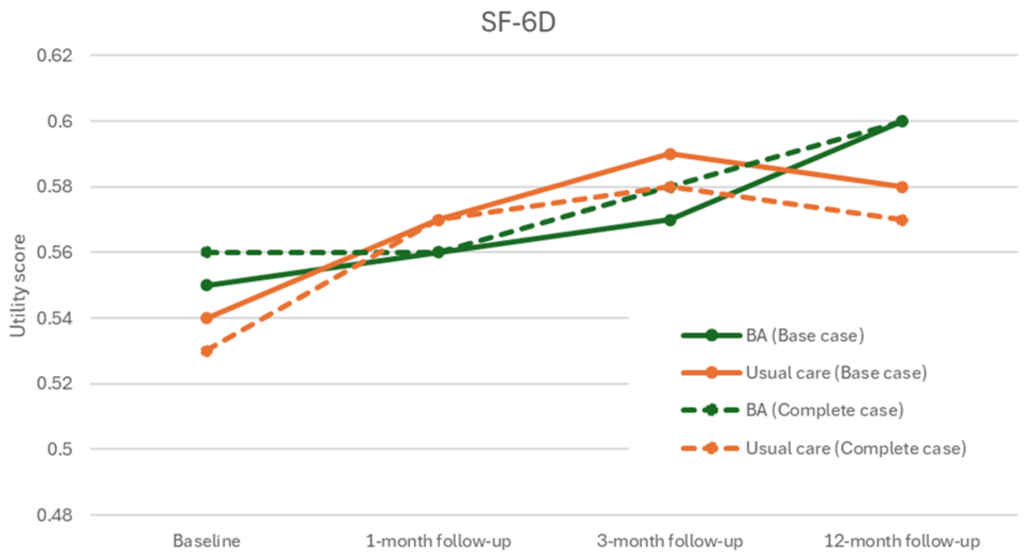

Appendix 9: EQ-5D-3L responses by trial arm by data collection time point

BA (n=218)	Baseline			1 month			3 months			12 months		
	Level 1 n (%)	Level 2 n (%)	Level 3 n (%)	Level 1 n (%)	Level 2 n (%)	Level 3 n (%)	Level 1 n (%)	Level 2 n (%)	Level 3 n (%)	Level 1 n (%)	Level 2 n (%)	Level 3 n (%)
Mobility	83 (38)	134 (62)	1 (0)	59 (36)	105 (64)	-	72 (45)	89 (55)	-	48 (34)	87 (62)	5 (4)
Self-care	168 (77)	47 (22)	3 (1)	136 (83)	24 (15)	4 (2)	134 (83)	26 (16)	1 (1)	102 (78)	35 (25)	3 (2)
Usual activity	74 (34)	130 (60)	14 (6)	69 (42)	87 (53)	8 (5)	69 (43)	83 (52)	9 (6)	50 (36)	78 (56)	12 (9)
Pain/discomfort	50 (23)	143 (66)	25 (12)	44 (27)	99 (60)	21 (12)	43 (27)	97 (68)	21 (13)	30 (21)	83 (59)	27 (19)
Anxiety/depression	97 (45)	114 (52)	7 (3)	93 (57)	67 (49)	4 (2)	100 (62)	58 (36)	3 (2)	71 (51)	67 (48)	2 (1)
**Usual care (n=217)**	**Baseline**			**1 months**			**3 months**			**12 months**		
	**Level 1 n (%)**	**Level 2 n (%)**	**Level 3 n (%)**	**Level 1 n (%)**	**Level 2 n (%)**	**Level 3 n (%)**	**Level 1 n (%)**	**Level 2 n (%)**	**Level 3 n (%)**	**Level 1 n (%)**	**Level 2 n (%)**	**Level 3 n (%)**
Mobility	84 (39)	129 (60)	4 (2)	67 (36)	120 (64)	-	69 (36)	119 (63)	2 (1)	51 (30)	116 (69)	1 (1)
Self-care	163 (75)	51 (24)	3 (1)	141 (75)	45 (24)	1 (1)	149 (78)	40 (21)	1 (1)	121 (72)	45 (27)	2 (1)
Usual activity	94 (43)	111 (51)	12 (6)	76 (41)	97 (52)	14 (8)	81 (43)	99 (52)	10 (5)	52 (31)	101 (60)	15 (9)
Pain/discomfort	59 (27)	137 (63)	21 (10)	49 (26)	117 (63)	21 (11)	49 (26)	122 (64)	19 (10)	36 (21)	112 (67)	20 (12)
Anxiety/depression	102 (47)	105 (48)	10 (5)	108 (58)	71 (38)	8 (4)	105 (55)	80 (42)	5 (3)	103 (61)	61 (36)	4 (2)

Note: Level 1: none, Level 2: some, Level 3: extreme

Appendix 10: SF-6D responses by trial arm by data collection time point

BA (n=218)	Baseline					1 month				
	Level 1 n (%)	Level 2 n (%)	Level 3 n (%)	Level 4 n (%)	Level 5 n (%)	Level 1 n (%)	Level 2 n (%)	Level 3 n (%)	Level 4 n (%)	Level 5 n (%)
Physical functioning	55 (25)	80 (37)	83 (38)	-	-	34 (21)	65 (40)	65 (40)	-	-
Role limitations	12 (6)	44 (20)	15 (7)	147 (67)	-	9 (6)	37 (23)	17 (10)	100 (61)	-
Social functioning	70 (32)	37 (17)	48 (22)	39 (18)	24 (11)	60 (37)	26 (16)	35 (21)	26 (16)	17 (10)
Pain	39 (18)	42 (19)	41 (19)	60 (28)	36 (17)	26 (16)	37 (23)	31 (19)	50 (31)	20 (12)
Mental health	26 (12)	55 (25)	95 (44)	34 (16)	8 (4)	22 (13)	53 (33)	69 (42)	17 (10)	3 (2)
Vitality	1 (1)	16 (7)	64 (29)	71 (33)	66 (30)	-	18 (11)	46 (28)	49 (30)	51 (31)
**Usual care (n=217)**	**Baseline**					**1 month**				
	Level 1 n (%)	Level 2 n (%)	Level 3 n (%)	Level 4 n (%)	Level 5 n (%)	Level 1 n (%)	Level 2 n (%)	Level 3 n (%)	Level 4 n (%)	Level 5 n (%)
Physical functioning	58 (27)	69 (32)	90 (42)	-	-	41 (22)	76 (40)	72 (38)	-	-
Role limitations	8 (4)	43 (20)	13 (6)	152 (70)	-	16 (9)	47 (25)	13 (7)	113 (60)	-
Social functioning	67 (31)	34 (16)	58 (27)	41 (19)	17 (8)	69 (37)	32 (17)	43 (23)	22 (12)	23 (12)
Pain	38 (18)	43 (20)	45 (21)	63 (29)	28 (13)	38 (20)	41 (22)	35 (19)	50 (27)	25 (13)
Mental health	21 (10)	52 (24)	100 (46)	38 (18)	6 (3)	28 (15)	52 (28)	81 (43)	22 (12)	6 (3)
Vitality	-	21 (10)	59 (27)	76 (35)	61 (28)	7 (4)	17 (9)	47 (25)	61 (32)	57 (30)

Note: Level 1: not at all / none of the time, Level 2: A little bit / a little of the time, Level 3: A bit / some of the time, Level 4: Quite a lot / most of the time, Level 5: A lot / all of the time

BA (n=218)	3 months					12 months				
	Level 1 n (%)	Level 2 n (%)	Level 3 n (%)	Level 4 n (%)	Level 5 n (%)	Level 1 n (%)	Level 2 n (%)	Level 3 n (%)	Level 4 n (%)	Level 5 n (%)
Physical functioning	43 (27)	54 (33)	64 (40)	-	-	50 (36)	48 (34)	41 (30)	-	-
Role limitations	17 (11)	44 (27)	15 (9)	84 (53)	-	14 (10)	45 (33)	8 (6)	71 (51)	-
Social functioning	62 (39)	36 (22)	32 (20)	23 (14)	8 (5)	42 (30)	24 (17)	35 (25)	17 (12)	21 (15)
Pain	28 (17)	47 (29)	23 (14)	43 (27)	20 (12)	24 (17)	36 (26)	24 (17)	34 (25)	21 (15)
Mental health	36 (22)	57 (35)	52 (32)	15 (9)	1 (1)	33 (24)	41 (30)	46 (33)	17 (12)	1 (1)
Vitality	-	23 (14)	52 (32)	44 (27)	42 (26)	2 (1)	10 (7)	33 (24)	46 (33)	48 (35)
**Usual care (n=217)**	**3 months**					**12 months**				
	Level 1 n (%)	Level 2 n (%)	Level 3 n (%)	Level 4 n (%)	Level 5 n (%)	Level 1 n (%)	Level 2 n (%)	Level 3 n (%)	Level 1 n (%)	Level 2 n (%)
Physical functioning	54 (28)	71 (37)	65 (34)	-	-	62 (36)	44 (26)	64 (38)	-	-
Role limitations	17 (9)	54 (29)	14 (7)	104 (55)	-	12 (7)	57 (33)	11 (7)	90 (53)	-
Social functioning	72 (38)	25 (13)	39 (21)	34 (18)	18 (10)	58 (34)	30 (18)	40 (24)	20 (12)	20 (12)
Pain	34 (18)	50 (26)	33 (17)	52 (27)	21 (11)	31 (18)	39 (23)	37 (22)	39 (23)	23 (14)
Mental health	43 (23)	62 (33)	56 (29)	21 (11)	8 (4)	44 (26)	51 (30)	47 (28)	22 (13)	5 (3)
Vitality	2 (1)	20 (11)	57 (30)	50 (26)	61 (32)	3 (2)	17 (10)	45 (27)	55 (32)	49 (29)

Note: Level 1: not at all / none of the time, Level 2: A little bit / a little of the time, Level 3: A bit / some of the time, Level 4: Quite a lot / most of the time, Level 5: A lot / all of the time

Appendix 11: Results of sensitivity analyses

Scenario	Incremental costs (£)<br>Mean (95%CI)	Incremental QALYs<br>Mean (95% CI)	ICER	Probability of BA being cost-effective*
Base case	-62.34 (-239.70 to 120.44)	0.007 (-0.036 to 0.022)	Dominant	0.710
Scenario 1: complete case	-21.71 (-269.17 to 220.15)	0.002 (-0.037 to 0.039)	Dominant	0.553
Scenario 2: from societal perspective	-47.77 (-239.77 to 155.87)	0.007 (-0.022 to 0.036)	Dominant	0.686
Scenario 3: QALY measured by SF-6D	-62.34 (-239.70 to 120.44)	0.016 (-0.012 to 0.043)	Dominant	0.882
Scenario 4: Excluding training costs	-80.84 (-263.27 to 95.85)	0.006 (-0.025 to 0.037)	Dominant	0.700
Scenario 5: Removing high-volume cases	-138.90 (-163.06 to 182.35)	0.016 (-0.037 to 0.021)	Dominant	0.690

*against the willingness-to-way threshold of £20,000/QALY gained

Appendix 12: Cost-effectiveness planes of sensitivity analyses

The mean incremental cost and QALY estimates from the complete case were consistent with the base case scenario, resulting in a negative cost per QALY gained (Scenario 1). A similar trend was observed when costs were measured from a societal perspective (Scenario 2), when QALYs were measured by the SF-6D (Scenario 3), when training costs were treated as a one-off cost and excluded from the cost estimates based on an NHS/PSS perspective (Scenario 4), and when two high-volume cases were removed (Scenario 5).

Scenario 1: Complete case analysis

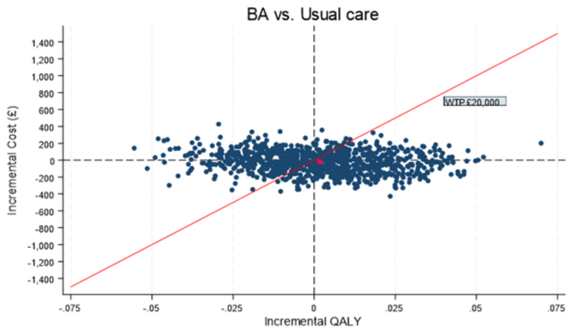

Scenario 2: Societal perspective

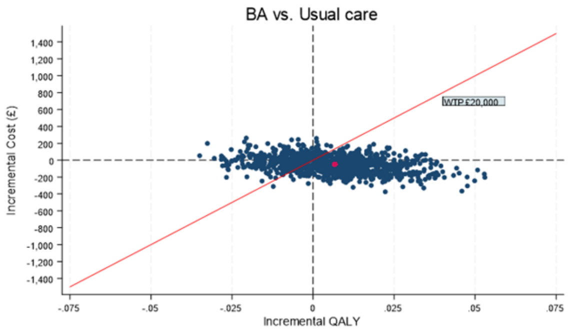

Scenario 3: SF-6D

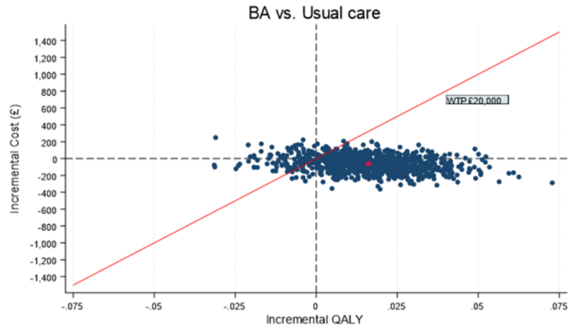

Scenario 4: Excluding training costs

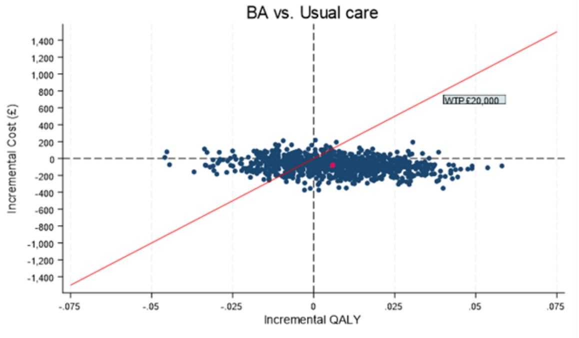

Scenario 5: Removing high-volume cases

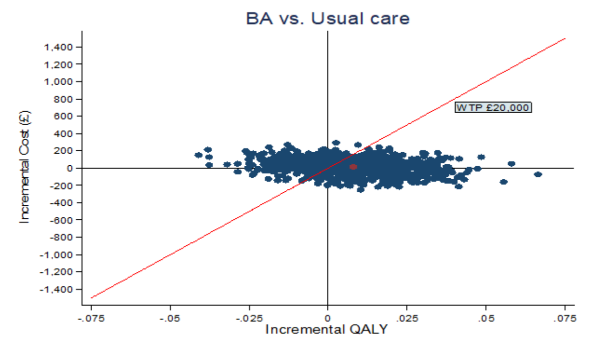

Supplement: online supplemental file 1 [file bmjment-28-1-s001.pdf]
